# Association Between Self-Reported Bruxism and Malocclusion in University Students: A Cross-Sectional Study

**DOI:** 10.2188/jea.JE20140180

**Published:** 2015-06-05

**Authors:** Kota Kataoka, Daisuke Ekuni, Shinsuke Mizutani, Takaaki Tomofuji, Tetsuji Azuma, Mayu Yamane, Yuya Kawabata, Yoshiaki Iwasaki, Manabu Morita

**Affiliations:** 1 Department of Preventive Dentistry, Okayama University Graduate School of Medicine, Dentistry and Pharmaceutical Sciences, Okayama, Japan; 1 岡山大学大学院医歯薬学総合研究科予防歯科学分野; 2 Advanced Research Center for Oral and Craniofacial Sciences, Okayama University Dental School, Okayama, Japan; 2 岡山大学歯学部先端領域研究センター; 3 Health Service Center, Okayama University, Okayama, Japan; 3 岡山大学保健管理センター

**Keywords:** bruxism, malocclusion, young adult, cross-sectional studies

## Abstract

**Objectives:**

Bruxism can result in temporomandibular disorders, oral pain, and tooth wear. However, it is unclear whether bruxism affects malocclusion. The aim of this study was to examine the association between self-reported bruxism and malocclusion in university students.

**Methods:**

Students (*n* = 1503; 896 men and 607 women) aged 18 and 19 years were examined. Malocclusion was defined using a modified version of the Index of Orthodontic Treatment Need. The presence of buccal mucosa ridging, tooth wear, dental impression on the tongue, palatal/mandibular torus, and the number of teeth present were recorded, as well as body mass index (BMI). Additional information regarding gender, awareness of bruxism, orthodontic treatment, and oral habits was collected via questionnaire.

**Results:**

The proportion of students with malocclusion was 32% (*n* = 481). The awareness of clenching in males with malocclusion was significantly higher than in those with normal occlusion (chi square test, *P* < 0.01). According to logistic regression analysis, the probability of malocclusion was significantly associated with awareness of clenching (odds ratio [OR] 2.19; 95% confidence interval [CI], 1.22–3.93) and underweight (BMI <18.5 kg/m^2^) (OR 1.89; 95% CI, 1.31–2.71) in males but not in females. In subgroup analyses, the probability of crowding was also significantly associated with awareness of clenching and underweight (*P* < 0.01) in males.

**Conclusions:**

Awareness of clenching and underweight were related to malocclusion (crowding) in university male students.

## Abstract (ja)

**目的：:**

ブラキシズムは、顎関節症、口腔内の痛みおよび歯の咬耗を引き起こしうることがわかっている。しかしながら、ブラキシズムが不正咬合に影響するかどうかはあまり知られていない。本研究の目的は、大学生を対象にブラキシズムの自覚と不正咬合との関連について検討することであった。

**方法：:**

大学入学時の歯科健診を受診した18歳、19歳の大学生1,503名（男性896名、女性607名）を対象とした。不正咬合は簡易版Index of Orthodontic Treatment Needを用いて評価した。また、頬粘膜圧痕、歯の咬耗、舌圧痕、口蓋／下顎隆起、現在歯数およびBody Mass Index （BMI）を検査した。自己記入式アンケート調査により、性別、ブラキシズムの自覚症状、歯科矯正経験の有無および口腔習癖の有無を調べた。

**結果：:**

不正咬合がみられる大学生の割合は32.0％（481名）であった。不正咬合を有する大学生では、正常咬合の者に比べて、ブラキシズムの自覚症状がある割合が有意に高かった（χ^2^検定、*P*<0.01）。ロジスティック回帰分析の結果では、男性において不正咬合の有無に関連する因子は、クレンチングの自覚（オッズ比 2.19、95%信頼区間 1.22-3.93、*P*<0.01）と低体重（BMI <18.5 kg/m^2^）（オッズ比 1.89、95%信頼区間1.31-2.71、*P*<0.01）であったが、女性ではそのような因子は認められなかった。不正咬合の種類別で分析した場合、男性では叢生とクレンチングの自覚および低体重との有意な関連があった（*P*<0.01）。

**結論：:**

男子大学生において、クレンチングの自覚および低体重は不正咬合（叢生）と関連があった。

## INTRODUCTION

Bruxism, defined as the parafunctional grinding of teeth, is an oral habit consisting of involuntary rhythmic or spasmodic non-functional gnashing, grinding, or clenching of the teeth other than chewing movements of the mandible.^1^ Although this definition is commonly used by dental professionals, there is no link to the sleep-wake state of the condition.^2^ Sleep bruxism is clearly defined as ‘an oral parafunction characterized by grinding or clenching of the teeth during sleep that is associated with excessive sleep arousal activity’.^3^ However, awake bruxism still lacks a clear definition.^2^

The prevalence of bruxism ranges widely, from 4% to 96%,^4^^–^^10^ because of differences in the bruxism types (unspecified, sleep, and awake), applied diagnostic methodology (questionnaires, oral history, and clinical examination), the presence or absence of comorbidities (eg, anxiety or temporomandibular disorder), and the characteristics of the study population.^2^ The prevalence of bruxism is higher in young adults than in the elderly.^6^^,^^11^^,^^12^ The etiology of bruxism remains controversial. Recent reviews suggest that bruxism is mainly regulated by pathophysiological and psychological factors, rather than morphological ones.^13^^,^^14^ Although some dentists suggest that malocclusion may cause bruxism, a recent review concluded that there is no evidence whatsoever for a causal relationship between bruxism and occlusion.^2^ Thus, the research focus is mainly on psychosocial,^15^^,^^16^ physiological/biological,^17^^–^^20^ and exogenous factors.^21^ However, the etiological factors for bruxism are still unclear, and the etiology is probably multifactorial.^15^^–^^22^

Intermittent bruxism, including clenching and grinding, is extremely common, but it usually poses no serious consequences for the oral structures. On the other hand, frequent bruxism can result in problems. Possible sequelae of bruxism include tooth wear, signs and symptoms of temporomandibular disorders, headache, toothache, mobile teeth, and various problems with dental restorations as well as with fixed and removable prostheses.^5^ However, whether possible consequences of bruxism also include malocclusion remains unclear.

Malocclusion is a developmental disorder of the maxillofacial system, and inflicts functional and esthetic disturbances. Malocclusion, including crowding, open bite, overjet, and crossbite, is associated with psychological stress as well as impaired oral health.^23^ The etiology of malocclusion has genetic and environmental components,^24^ and study of malocclusion is essential for the success of orthodontic treatment, since a prerequisite for correction is the elimination of the causes of malocclusion.^25^ Analysis of factors related to the causes of malocclusion is important for planning public health policies aimed at preventing and clinically intercepting the health problem.^26^ Since excessive force exerted by bruxism can lead to tooth movement,^27^ bruxism may result in malocclusion. Therefore, we hypothesized that bruxism may be a risk factor of malocclusion. The aim of this study was to investigate the association between bruxism and malocclusion in university students.

## MATERIALS AND METHODS

### Study population

Of 2303 first-year students who underwent a general health examination at the Health Service Center of Okayama University in April 2013, 2205 students volunteered to receive an oral examination and answered the questionnaire described below. The general health examination is mandatory for first-year students in all departments in the university. We excluded 702 participants who were ≥20 years old (*n* = 113), who had received or were currently undergoing orthodontic treatment (*n* = 431), or who had provided incomplete data in their questionnaires (*n* = 158). Therefore, data from 1503 students (896 men and 607 women) were analyzed. The study was approved by the Ethics Committee of Okayama University Graduate School of Medicine, Dentistry and Pharmaceutical Sciences. Written consent was obtained from all participants.

### Questionnaire

We mailed a questionnaire to the participants before the health examination. Besides gender, age, and general condition, the questionnaire included questions on awareness of bruxism and oral habits. Questions to identify awareness of bruxism included the following: During the past 3 months, 1) Has anyone heard you grinding your teeth at night?; 2) Is your jaw ever fatigued or sore on waking in the morning?; 3) Are your teeth or gums ever sore on waking in the morning?; 4) Do you ever experience temporal headache on waking in the morning?; 5) Are you ever aware of grinding your teeth during the day?; and 6) Are you ever aware of clenching your teeth during the day?^3^^,^^28^^–^^30^ Each question was answered by selecting a frequency (frequently, sometimes, rarely, or never). We combined “frequently” and “sometimes” responses into a single category of positive awareness and rarely and never responses into a single category of negative awareness. The validity and reliability of the questionnaire has been already confirmed as useful for evaluating bruxism.^3^^,^^28^^–^^30^ For oral habits, answers were given by participants in a “yes/no” format as follows: biting fingernail/pens/pencils, biting mucosa of cheeks/lips, and gum chewing.^31^^–^^33^

### Assessment of body mass index

In the general health examination, the height and body weight of participants were measured by the university’s public health nurses using the Tanita body fat analyser (Model No. BF-220; Tanita Co., Tokyo, Japan). Since body mass index (BMI) may be related to jaw growth, BMI was computed as weight in kilograms divided by height in meters squared.^34^ For this analysis, categories of BMI were calculated based on the accepted cut-off values of underweight (BMI <18.5 kg/m^2^), normal weight (18.5–24.9 kg/m^2^), overweight (25–29.9 kg/m^2^), and obese (≥30 kg/m^2^).^35^

### Oral examination

Five dentists (S.M., D.E., T.A., M.Y., and K.K.) examined the study participants. In addition to the number of teeth present, occlusal tooth wear was assessed using the occlusal tooth wear index: score 0 = no loss of occlusal enamel surface characteristics; score 1 = loss of occlusal enamel surface characteristics; score 2 = loss of occlusal enamel, exposing dentine for less than 1/3 of the surface/incisal loss of enamel and minimal dentine exposure; score 3 = loss of occlusal enamel, exposing dentine for more than 1/3 of the surface/incisal loss of enamel and substantial loss of dentine; and score 4 = complete loss of occlusal/incisal enamel, pulp exposure or exposure of secondary dentine.^36^

For malocclusion, a modified version of the Index of Orthodontic Treatment Need (IOTN) was used for each participant.^23^ The modified IOTN that we used does not define a definite aesthetic need for treatment (AC grades 8, 9, and 10). However, previous studies suggested that our modified version of IOTN without a definite aesthetic need for treatment,^23^^,^^37^ as well as the modified IOTN (the original reference)^38^ are useful for screening malocclusion by non-specialists in oral health surveys. The dental health component of the modified IOTN consists of a two-grade scale (0 = no definite need for orthodontic treatment [normal occlusion group] and 1 = definite need for orthodontic treatment [malocclusion group]). Further, for subgroup analysis, the type of malocclusion (crowding, overjet, overbite, crossbites, and missing teeth) was recorded using a CPI probe (YDM, Tokyo, Japan).

The diagnostic criterion of buccal mucosa ridging or tongue indentation was defined as a linear thickening at the level where the teeth occlude on the buccal mucosa or tongue, respectively.^30^ In buccal mucosa ridging, the range (none, partial, and widespread) was also evaluated.

The presence or absence of palatal torus and mandibular torus was examined.^39^ Palatal torus was assessed as present when a painless bony swelling was visualized or palpated in the middle of the hard palate.^40^ Mandibular torus was assessed as present when a painless bony outgrowth in the lingual area of the mandible was visualized or palpated.

In a preliminary check, each kappa value (for occlusal tooth wear, malocclusion, buccal mucosa ridging, dental impression on the tongue, palatal torus, and mandibular torus) was more than 0.80.

### Statistical analyses

Unpaired *t* or chi-squared tests were used to determine whether there were any significant differences (*P* < 0.05) between men and women and between the normal occlusion and malocclusion groups. Odds ratios (ORs) and 95% confidence intervals (CIs) were calculated using a series of logistic regression models. Presence of malocclusion or crowding was used as a dependent variable. Based on the binary analyses, BMI category and clenching during the day were added as independent variables on multivariate analysis. In the multivariate analyses, we excluded tooth wear, buccal mucosa ridging, tongue indentation, palatal torus, and mandibular torus from the independent valuables because most of these items still contain vague factors and the validity has not been confirmed.^30^^,^^41^ A statistical program (PASW version 18.0; IBM, Tokyo, Japan) was used for statistical analyses.

## RESULTS

Table 1 shows the characteristics of participants. It was observed that 32.0% of participants had malocclusion (*n* = 481), with the prevalence of malocclusion in females being significantly higher than that in males (*P* < 0.05). Since there were significant differences in other clinical parameters, awareness of bruxism, and oral habits between males and females (*P* < 0.05), further analyses were performed in each gender.

**Table 1.** Characteristics of participants

Parameter	Males (*n* = 896)	Females (*n* = 607)	Total (*n* = 1503)	*P* value^a^
BMI (kg/m^2^)	21.2 ± 3.1	20.5 ± 2.5	20.9 ± 2.9	<0.001
BMI category				0.001
Underweight (BMI <18.5 kg/m^2^)	157 (17.5)	130 (21.4)	287 (19.1)	
Normal weight (BMI 18.5–24.9 kg/m^2^)	651 (72.7)	450 (74.1)	1101 (73.3)	
Overweight (BMI 25–29.9 kg/m^2^)	78 (8.7)	23 (3.8)	101 (6.7)	
Obesity (BMI ≥30 kg/m^2^)	10 (1.1)	4 (0.7)	14 (0.9)	
Number of teeth present	28.7 ± 1.3	28.4 ± 1.1	28.5 ± 1.3	<0.001
Maximum occlusal tooth wear index score				0.007
0	211 (23.5)	189 (31.1)	400 (26.6)	
1	499 (55.7)	297 (48.9)	796 (53.0)	
2	168 (18.8)	105 (17.3)	273 (18.2)	
3	18 (2.0)	16 (2.6)	34 (2.3)	
4	0 (0.0)	0 (0.0)	0 (0.0)	
Buccal mucosa ridging				<0.001
No	379 (42.3)	178 (29.3)	557 (37.1)	
Partial	380 (42.4)	320 (52.7)	700 (46.6)	
Widespread	137 (15.3)	109 (18.0)	246 (16.4)	
Tongue indentation	249 (27.8)	207 (34.1)	456 (30.3)	0.009
Palatal torus	6 (0.7)	14 (2.3)	20 (1.3)	0.007
Mandibular torus	146 (16.3)	92 (15.2)	238 (15.8)	0.553
Malocclusion	268 (29.9)	213 (35.1)	481 (32.0)	0.025
Crowding	191 (21.3)	142 (23.4)	333 (22.2)	0.341
Overjet	67 (7.5)	56 (9.2)	123 (8.2)	0.102
Overbite	5 (0.6)	1 (0.2)	6 (0.4)	0.235
Crossbites	20 (2.2)	14 (2.3)	34 (2.3)	0.924
Missing teeth	18 (2.0)	7 (1.2)	25 (1.7)	0.203
Awareness of bruxism				
Jaw fatigue on waking in the morning	23 (2.6)	21 (3.5)	44 (2.9)	0.314
Sore teeth or gums on waking in the morning	20 (2.2)	16 (2.6)	36 (2.4)	0.615
Headache on waking in the morning	57 (6.4)	52 (8.6)	109 (7.3)	0.106
Grinding during the day	13 (1.5)	20 (3.3)	33 (2.2)	0.017
Clenching during the day	49 (5.5)	76 (12.5)	125 (8.3)	<0.001
Sleep bruxism	49 (5.5)	52 (8.6)	101 (6.7)	0.019
Oral habits				
Biting fingernail/pens/pencils	91 (10.2)	39 (6.4)	130 (8.6)	0.012
Biting mucosa of cheeks/lips	164 (18.3)	114 (18.8)	278 (18.5)	0.815
Gum chewing	134 (15.0)	46 (7.6)	180 (12.0)	<0.001

BMI, body mass index.

All values are reported as number (%) except for BMI, which is reported as mean ± standard deviation.

^a^*t* test or χ^2^ test.

Table 2 shows the association between malocclusion and related factors in males. There were significant differences in awareness of daytime clenching, buccal mucosa ridging, tongue indentation, mandibular torus, and BMI between the malocclusion and normal occlusion groups (*P* < 0.05).

**Table 2.** Association between malocclusion and related factors in males

Parameter	Normal occlusion (*n* = 628)	Malocclusion (*n* = 268)	*P* value^a^
BMI (kg/m^2^)	21.3 ± 3.1	20.8 ± 3.0	0.022
BMI category			0.006
Underweight (BMI <18.5 kg/m^2^)	92 (14.6)	65 (24.3)	
Normal weight (BMI 18.5–24.9 kg/m^2^)	473 (75.3)	178 (66.4)	
Overweight (BMI 25–29.9 kg/m^2^)	55 (8.8)	23 (8.6)	
Obesity (BMI ≥30 kg/m^2^)	8 (1.3)	2 (0.7)	
Number of teeth present	28.7 ± 1.3	28.6 ± 1.3	0.398
Maximum occlusal tooth wear index score			0.062
0	150 (23.9)	61 (22.8)	
1	358 (57.0)	141 (52.6)	
2	112 (17.8)	56 (20.9)	
3	8 (1.3)	10 (3.7)	
4	0 (0.0)	0 (0.0)	
Buccal mucosa ridging			<0.001
No	295 (47.0)	84 (31.3)	
Partial	249 (39.6)	131 (48.9)	
Widespread	84 (13.4)	53 (19.8)	
Tongue indentation	157 (25.0)	92 (34.3)	0.004
Palatal torus	4 (0.6)	2 (0.7)	0.854
Mandibular torus	80 (12.7)	66 (24.6)	<0.001
Awareness of bruxism			
Jaw fatigue on waking in the morning	15 (2.4)	8 (3.0)	0.605
Sore teeth or gums on waking in the morning	12 (1.9)	8 (3.0)	0.319
Headache on waking in the morning	46 (7.3)	11 (4.1)	0.071
Grinding during the day	10 (1.6)	3 (1.1)	0.588
Clenching during the day	26 (4.1)	23 (8.6)	0.007
Sleep bruxism	34 (5.4)	15 (5.6)	0.912
Oral habits			
Biting fingernail/pens/pencils	67 (10.7)	24 (9.0)	0.437
Biting mucosa of cheeks/lips	106 (16.9)	58 (21.6)	0.091
Gum chewing	96 (15.3)	38 (14.2)	0.670

BMI, body mass index.

All values are reported as number (%) except for BMI, which is reported as mean ± standard deviation.

^a^*t* test or χ^2^ test.

In females, there were significant differences in maximum occlusal tooth wear index score, buccal mucosa ridging, tongue indentation, palatal torus, and mandibular torus between the malocclusion and normal occlusion groups (*P* < 0.05) (Table 3). However, significant between-group differences in awareness of daytime clenching and BMI were not observed in females.

**Table 3.** Association between malocclusion and related factors in females

Parameter	Normal occlusion (*n* = 394)	Malocclusion (*n* = 213)	*P* value^a^
BMI (kg/m^2^)	20.5 ± 2.4	20.5 ± 2.7	0.990
BMI category			0.252
Underweight (BMI <18.5 kg/m^2^)	81 (20.6)	49 (23.0)	
Normal weight (BMI 18.5–24.9 kg/m^2^)	295 (74.9)	155 (72.8)	
Overweight (BMI 25–29.9 kg/m^2^)	17 (4.3)	6 (2.8)	
Obesity (BMI ≥30 kg/m^2^)	1 (0.3)	3 (1.4)	
Number of teeth present	28.4 ± 1.1	28.5 ± 1.2	0.369
Maximum occlusal tooth wear index score			0.014
0	114 (28.9)	75 (35.3)	
1	208 (52.8)	89 (41.8)	
2	66 (16.8)	39 (18.3)	
3	6 (1.5)	10 (4.7)	
4	0 (0.0)	0 (0.0)	
Buccal mucosa ridging			0.002
No	134 (34.0)	44 (20.7)	
Partial	190 (48.2)	130 (61.0)	
Widespread	70 (17.8)	39 (18.3)	
Tongue indentation	118 (29.9)	89 (41.8)	0.003
Palatal torus	4 (1.0)	10 (4.7)	0.004
Mandibular torus	38 (9.6)	54 (25.4)	<0.001
Awareness of bruxism			
Jaw fatigue on waking in the morning	13 (3.3)	8 (3.8)	0.769
Sore teeth or gums on waking in the morning	10 (2.5)	6 (2.8)	0.838
Headache on waking in the morning	33 (8.4)	19 (8.9)	0.819
Grinding during the day	11 (2.8)	9 (4.2)	0.345
Clenching during the day	50 (12.7)	26 (12.2)	0.864
Sleep bruxism	37 (9.4)	15 (7.0)	0.324
Oral habits			
Biting fingernail/pens/pencils	25 (6.3)	14 (6.6)	0.913
Biting mucosa of cheeks/lips	82 (20.8)	32 (15.0)	0.081
Gum chewing	28 (7.1)	18 (8.5)	0.550

BMI, body mass index.

All values are reported as number (%) except for BMI, which is reported as mean ± standard deviation.

^a^*t* test or χ^2^ test.

On logistic regression analyses, the risk of malocclusion was significantly related to underweight (BMI <18.5 kg/m^2^) and clenching during the day in males (*P* < 0.01) (Table 4). However, there was no significant association between malocclusion and any of the parameters in females.

**Table 4.** Adjusted odds ratios and 95% confidence intervals for malocclusion

Parameter		Odds ratio	95% CI^a^	*P* value
Males				

BMI category	Normal weight (BMI 18.5–24.9 kg/m^2^)	1		
	Underweight (BMI <18.5 kg/m^2^)	1.89	1.31–2.71	0.001
	Overweight/obesity (BMI ≥25 kg/m^2^)	1.08	0.66–1.77	0.771
Clenching during the day	−	1		
	+	2.19	1.22–3.93	0.009

Females				

BMI category	Normal weight (BMI 18.5–24.9 kg/m^2^)	1		
	Underweight (BMI <18.5 kg/m^2^)	1.17	0.78–1.76	0.438
	Overweight/obesity (BMI ≥25 kg/m^2^)	0.94	0.41–2.15	0.888
Clenching during the day	−	1		
	+	1.06	0.64–1.74	0.833

BMI, body mass index; CI, confidence interval.

^a^Adjusted for BMI and clenching.

Because of the small number of participants with overbite, crossbites, and missing teeth, further subgroup analysis was performed only between normal occlusion and crowding groups and between normal occlusion and overjet groups.

Table 5 shows the association between crowding and related factors in males. There were significant differences in BMI, buccal mucosa ridging, tongue indentation, awareness of daytime clenching, and mandibular torus between the crowding and normal occlusion groups (*P* < 0.05).

**Table 5.** Association between crowding and related factors in males

Parameter	Normal occlusion (*n* = 628)	Crowding (*n* = 191)	*P* value^a^
BMI (kg/m^2^)	21.3 ± 3.1	20.7 ± 3.1	0.016
BMI category			0.001
Underweight (BMI <18.5 kg/m^2^)	92 (14.6)	51 (26.7)	
Normal weight (BMI 18.5–24.9 kg/m^2^)	473 (75.3)	121 (63.4)	
Overweight (BMI 25–29.9 kg/m^2^)	55 (8.8)	18 (9.4)	
Obesity (BMI ≥30 kg/m^2^)	8 (1.3)	1 (0.5)	
Number of teeth present	28.7 ± 1.3	28.6 ± 1.2	0.317
Maximum occlusal tooth wear index score			0.129
0	150 (23.9)	45 (23.6)	
1	358 (57.0)	100 (52.4)	
2	112 (17.8)	39 (20.4)	
3	8 (1.3)	7 (3.7)	
4	0 (0.0)	0 (0.0)	
Buccal mucosa ridging			<0.001
No	295 (47.0)	58 (30.4)	
Partial	249 (39.6)	94 (49.2)	
Widespread	84 (13.4)	39 (20.4)	
Dental impression on the tongue	157 (25.0)	67 (35.1)	0.006
Palatal torus	4 (0.6)	2 (1.0)	0.561
Mandibular torus	80 (12.7)	49 (25.7)	<0.001
Awareness of bruxism			
Jaw fatigue on waking in the morning	15 (2.4)	8 (4.2)	0.187
Sore teeth or gums on waking in the morning	12 (1.9)	7 (3.7)	0.158
Headache on waking in the morning	46 (7.3)	9 (4.7)	0.206
Grinding during the day	10 (1.6)	3 (1.6)	0.983
Clenching during the day	26 (4.1)	19 (9.9)	0.002
Sleep bruxism	34 (5.4)	12 (6.3)	0.648
Oral habits			
Biting fingernail/pens/pencils	67 (10.7)	16 (8.4)	0.358
Biting mucosa of cheeks/lips	106 (16.9)	44 (23.0)	0.054
Gum chewing	96 (15.3)	29 (15.2)	0.972

BMI, body mass index.

All values are reported as number (%) except for BMI, which is reported as mean ± standard deviation.

^a^*t* test or χ^2^ test.

In females, there were significant differences in number of teeth present, maximum occlusal tooth wear index score, buccal mucosa ridging, tongue indentation, palatal torus, and mandibular torus between the crowding and normal occlusion groups (*P* < 0.05) (Table 6). However, significant between-group differences in awareness of daytime clenching and BMI were not observed in females.

**Table 6.** Association between crowding and related factors in females

Parameter	Normal occlusion (*n* = 394)	Crowding (*n* = 142)	*P* value^a^
BMI (kg/m^2^)	20.5 ± 2.4	20.5 ± 2.5	0.844
BMI category			0.578
Underweight (BMI <18.5 kg/m^2^)	81 (20.6)	29 (20.4)	
Normal weight (BMI 18.5–24.9 kg/m^2^)	295 (74.9)	109 (76.8)	
Overweight (BMI 25–29.9 kg/m^2^)	17 (4.3)	3 (2.1)	
Obesity (BMI ≥30 kg/m^2^)	1 (0.3)	1 (0.7)	
Number of teeth present	28.4 ± 1.1	28.7 ± 1.2	0.017
Maximum occlusal tooth wear index score			0.008
0	114 (28.9)	44 (31.0)	
1	208 (52.8)	60 (42.3)	
2	66 (16.8)	29 (20.4)	
3	6 (1.5)	9 (6.3)	
4	0 (0.0)	0 (0.0)	
Buccal mucosa ridging			0.006
No	134 (34.0)	31 (21.8)	
Partial	190 (48.2)	83 (58.5)	
Widespread	70 (17.8)	28 (19.7)	
Dental impression on the tongue	118 (29.9)	65 (45.8)	0.001
Palatal torus	4 (1.0)	8 (5.6)	0.001
Mandibular torus	38 (9.6)	40 (28.2)	<0.001
Awareness of bruxism			
Jaw fatigue on waking in the morning	13 (3.3)	7 (4.9)	0.380
Sore teeth or gums on waking in the morning	10 (2.5)	4 (2.8)	0.858
Headache on waking in the morning	33 (8.4)	16 (11.3)	0.305
Grinding during the day	11 (2.8)	6 (4.2)	0.403
Clenching during the day	50 (12.7)	17 (13.0)	0.824
Sleep bruxism	37 (9.4)	11 (7.7)	0.556
Oral habits			
Biting fingernail/pens/pencils	25 (6.3)	8 (5.6)	0.762
Biting mucosa of cheeks/lips	82 (20.8)	19 (13.4)	0.052
Gum chewing	28 (7.1)	11 (7.7)	0.801

BMI, body mass index.

All values are reported as number (%) except for BMI, which is reported as mean ± standard deviation.

^a^*t* test or χ^2^ test.

On logistic regression analyses, the risk of crowding was significantly related to underweight (BMI <18.5 kg/m^2^) and clenching during the day in males (*P* < 0.01) (Table 7). In females, the risk of crowding was significantly related to the number of teeth present (*P* < 0.01) (Table 7).

**Table 7.** Adjusted odds ratios and 95% confidence intervals for crowding

Parameter		Odds ratio	95% CI^a^	*P* value
Males				

BMI category	Normal weight (BMI 18.5–24.9 kg/m^2^)	1		
	Underweight (BMI <18.5 kg/m^2^)	2.21	1.48–3.30	<0.001
	Overweight/obesity (BMI ≥25 kg/m^2^)	1.25	0.72–2.18	0.431
Number of teeth present		0.916	0.80–1.05	0.194
Clenching during the day	−	1		
	+	2.71	1.45–5.07	0.002

Females				

BMI category	Normal weight (BMI 18.5–24.9 kg/m^2^)	1		
	Underweight (BMI <18.5 kg/m^2^)	1.04	0.64–1.69	0.879
	Overweight/obesity (BMI ≥25 kg/m^2^)	0.53	0.17–1.62	0.266
Number of teeth present		1.24	1.06–1.46	0.009
Clenching during the day	−	1		
	+	0.96	0.53–1.73	0.886

BMI, body mass index; CI, confidence interval.

^a^Adjusted for BMI and clenching.

Table 8 and Table 9 show the association between overjet and related factors in males and females, respectively. There were no significant differences in any factor between the overjet and normal occlusion groups (*P* > 0.05).

**Table 8.** Association between overjet and related factors in males

Parameter	Normal occlusion (*n* = 628)	Overjet (*n* = 67)	*P* value^a^
BMI (kg/m^2^)	21.3 ± 3.1	20.9 ± 3.0	0.340
BMI category			0.235
Underweight (BMI <18.5 kg/m^2^)	92 (14.6)	16 (23.9)	
Normal weight (BMI 18.5–24.9 kg/m^2^)	473 (75.3)	46 (68.7)	
Overweight (BMI 25–29.9 kg/m^2^)	55 (8.8)	4 (6.0)	
Obesity (BMI ≥30 kg/m^2^)	8 (1.3)	1 (1.5)	
Number of teeth present	28.7 ± 1.3	28.6 ± 1.5	0.641
Maximum occlusal tooth wear index score			0.541
0	150 (23.9)	12 (17.9)	
1	358 (57.0)	38 (56.7)	
2	112 (17.8)	16 (23.9)	
3	8 (1.3)	1 (1.5)	
4	0 (0.0)	0 (0.0)	
Buccal mucosa ridging			0.283
No	295 (47.0)	25 (37.3)	
Partial	249 (39.6)	30 (44.8)	
Widespread	84 (13.4)	12 (17.9)	
Dental impression on the tongue	157 (25.0)	17 (25.4)	0.947
Palatal torus	4 (0.6)	0 (0.0)	0.512
Mandibular torus	80 (12.7)	12 (17.9)	0.235
Awareness of bruxism			
Jaw fatigue on waking in the morning	15 (2.4)	1 (1.5)	0.642
Sore teeth or gums on waking in the morning	12 (1.9)	1 (1.5)	0.810
Headache on waking in the morning	46 (7.3)	3 (4.5)	0.387
Grinding during the day	10 (1.6)	0 (0.0)	0.298
Clenching during the day	26 (4.1)	5 (7.5)	0.210
Sleep bruxism	34 (5.4)	4 (6.0)	0.849
Oral habits			
Biting fingernail/pens/pencils	67 (10.7)	4 (6.0)	0.227
Biting mucosa of cheeks/lips	106 (16.9)	16 (23.9)	0.152
Gum chewing	96 (15.3)	9 (13.4)	0.687

BMI, body mass index.

All values are reported as number (%) except for BMI, which is reported as mean ± standard deviation.

^a^*t* test or χ^2^ test.

**Table 9.** Association between overjet and related factors in females

Parameter	Normal occlusion (*n* = 394)	Overjet (*n* = 56)	*P* value^a^
BMI (kg/m^2^)	20.5 ± 2.4	19.9 ± 2.0	0.065
BMI category			0.118
Underweight (BMI <18.5 kg/m^2^)	81 (20.6)	18 (32.1)	
Normal weight (BMI 18.5–24.9 kg/m^2^)	295 (74.9)	38 (67.9)	
Overweight (BMI 25–29.9 kg/m^2^)	17 (4.3)	0 (0.0)	
Obesity (BMI ≥30 kg/m^2^)	1 (0.3)	0 (0.0)	
Number of teeth present	28.4 ± 1.1	28.2 ± 1.2	0.297
Maximum occlusal tooth wear index score			0.618
0	114 (28.9)	21 (37.5)	
1	208 (52.8)	26 (46.4)	
2	66 (16.8)	8 (14.3)	
3	6 (1.5)	1 (1.8)	
4	0 (0.0)	0 (0.0)	
Buccal mucosa ridging			0.298
No	134 (34.0)	14 (25.0)	
Partial	190 (48.2)	33 (58.9)	
Widespread	70 (17.8)	9 (16.1)	
Dental impression on the tongue	118 (29.9)	16 (28.6)	0.833
Palatal torus	4 (1.0)	2 (3.6)	0.119
Mandibular torus	38 (9.6)	9 (16.1)	0.141
Awareness of bruxism			
Jaw fatigue on waking in the morning	13 (3.3)	1 (1.8)	0.542
Sore teeth or gums on waking in the morning	10 (2.5)	3 (5.4)	0.239
Headache on waking in the morning	33 (8.4)	4 (7.1)	0.753
Grinding during the day	11 (2.8)	3 (5.4)	0.301
Clenching during the day	50 (12.7)	6 (10.7)	0.675
Sleep bruxism	37 (9.4)	4 (7.1)	0.584
Oral habits			
Biting fingernail/pens/pencils	25 (6.3)	3 (5.4)	0.775
Biting mucosa of cheeks/lips	82 (20.8)	10 (17.9)	0.608
Gum chewing	28 (7.1)	5 (8.9)	0.625

BMI, body mass index.

All values are reported as number (%) except for BMI, which is reported as mean ± standard deviation.

^a^*t* test or χ^2^ test.

## DISCUSSION

This is the first large-scale cross-sectional study to examine the association between malocclusion and awareness of bruxism in young adults based on the new hypothesis that bruxism may contribute to malocclusion. The results revealed that the risk of malocclusion, especially crowding, was significantly related to clenching during the day in males. Because the force of bruxism can lead to tooth movement^27^ and the tightness of proximal tooth contact is increased by clenching,^42^ clenching during the day may be a risk factor of malocclusion (crowding) through tooth movement in males.

The relationship between bruxism and occlusion has been investigated in dentistry for a long time but has remained poorly understood.^2^ Although some dentists suggest that malocclusion may cause bruxism, a recent review concluded that there is no evidence whatsoever for a causal relationship between bruxism and occlusion.^2^ Therefore, we hypothesized an inverse pathway, in which bruxism may be a risk factor of malocclusion. Our results support this hypothesis. However, the present study was a cross-sectional study; a prospective cohort or intervention study may provide information beyond what we present here.

In the present study, underweight (BMI <18.5 kg/m^2^) was also significantly related to malocclusion in males. There is a positive correlation between BMI and cervical vertebral maturation.^43^ Skeletal maturation is also related to malocclusion.^44^ Thus, skeletal prematurity with underweight may be related to malocclusion in males. However, the opposite pathway might be involved in the relationship. Malocclusion negatively affects subjects’ ability to process and break down foods, which then attenuates masticatory performance.^45^ In elderly people, masticatory performance is positively related to BMI.^46^ Malocclusion might induce underweight in young adults via reduction of masticatory performance. Further longitudinal studies are required to investigate the relationship between malocclusion and BMI.

In females, there was no significant association between malocclusion and awareness of bruxism. Although the reason for this lack of observed association is unclear, a possible mechanism may be as follows: in young adults, bite force in males is greater than that in females.^47^^–^^49^ Bruxism in females may therefore have smaller effects on tooth movement than that in males because of gender differences in bite force. Thus, in females, the association between bruxism and malocclusion may not have been obvious in this study. However, this possibility cannot explain the fact that malocclusion was more prevalent in females than males, which suggests that there are other risk factors of malocclusion in females, such as genetic factors. Further studies are required to investigate the gender difference.

Malocclusion can induce psychological stress and is associated with self-rated poor oral health in university students.^23^^,^^37^ Therefore, evaluation of bruxism might be required in university students for the prevention of malocclusion so that, for people with awareness of clenching, attempts can be made to avoid their clenching habit. In Japanese schools/universities, health examinations are performed on a regular basis, according to school health law. Since early control of the risk factors of malocclusion is essential for prevention in younger populations, monitoring of awareness of bruxism during regular health examinations might be useful.

We also investigated the association between awareness of bruxism and its related clinical factors, including maximum occlusal tooth wear index score, buccal mucosa ridging, tongue indentation, palatal torus, and mandibular torus. Females who had sore teeth and gums or jaw fatigue on waking in the morning had a higher prevalence of palatal torus than those who did not (12.5% vs 2.0% [*P* < 0.01] and 9.5% vs 2.0% [*P* < 0.01], respectively). Our results were supported by previous studies.^50^^–^^52^ On the other hand, other clinical factors, such as tooth wear, buccal mucosa ridging, tongue indentation, and mandibular torus, were not significantly related to bruxism. Bruxism seems to be one of the contributory factors for the development of tooth wear, oral tori, buccal mucosa ridging, and tongue indentation, and these items are sometimes used for clinical diagnosis of bruxism.^41^ However, most of these items still contain vague factors and their validity for diagnosing bruxism has not been confirmed.^41^

We used a self-administered questionnaire of bruxism because it can be applied to a large population and is convenient for clinicians and researchers to assess the presence or absence of bruxism, especially in epidemiological studies.^5^^,^^41^^,^^53^ On the other hand, a review suggests that the use of self-reports alone to assess the presence or absence of bruxism is scientifically unreliable.^41^ Actually, the prevalence of bruxism by self-reported questionnaires varies substantially (4%–40%),^10^ which is thought to be due to the limitation of self-reports.^41^ This can be considered a limitation in our study, since we did not measure bruxism activity directly using intra-oral appliances.

Our study has other limitations. First, all participants were recruited from among students at Okayama University, which may limit the ability to extrapolate these findings to the general population of young adults. However, based on Ministry of Education, Culture, Sports, Science and Technology in Japan, the proportion of students who went on to universities is about 50%. Further, the prevalence of malocclusion (32.1%) and awareness of clenching during the day (8.3%) were within the ranges reported in previous studies.^10^^,^^23^ Thus, there may be potential for generalization. Second, we did not examine other possible confounders, such as non-nutritive sucking,^54^ habitual mouth breathing,^55^ early loss of primary teeth,^56^ genetic factors, and occlusal force.^57^

In conclusion, this study revealed that awareness of clenching and underweight were related to malocclusion (crowding) in university male students. These findings suggest that clenching and BMI may be considered in screening for malocclusion risk in young male adults.

## ONLINE ONLY MATERIAL

Abstract in Japanese.
