# Supplementary material for: Association Between Self-Reported Bruxism and Malocclusion in University Students: A Cross-Sectional Study
Source: J Epidemiol. 2015 Jun 5;25(6):423–30. doi: 10.2188/jea.JE20140180 (PMC4444496; doi:10.2188/jea.JE20140180)
Supplement: Abstract in Japanese. [file je-25-423-s001.pdf]

・大学生におけるブラキシズムの自覚と不正咬合との関連についての横断調査

・片岡広太[1]、江國大輔[1]、水谷慎介[1]、友藤孝明[1,2]、東　哲司[1]、山根真由[1]、
川端勇也[1]、岩崎良章[3]、森田　学[1]
[1]岡山大学大学院医歯薬学総合研究科予防歯科学分野
[2]岡山大学歯学部先端領域研究センター
[3]岡山大学保健管理センター

目的：ブラキシズムは、顎関節症、口腔内の痛みおよび歯の咬耗を引き起こしうることがわかっている。しかしながら、ブラキシズムが不正咬合に影響するかどうかはあまり知られていない。本研究の目的は、大学生を対象にブラキシズムの自覚と不正咬合との関連について検討することであった。

方法：大学入学時の歯科健診を受診した 18 歳、19 歳の大学生 1,503 名（男性 896 名、女性 607 名）を対象とした。不正咬合は簡易版 Index of Orthodontic Treatment Need を用いて評価した。また、頬粘膜圧痕、歯の咬耗、舌圧痕、口蓋／下顎隆起、現在歯数および Body Mass Index （BMI）を検査した。自己記入式アンケート調査により、性別、ブラキシズムの自覚症状、歯科矯正経験の有無および口腔習癖の有無を調べた。

結果：不正咬合がみられる大学生の割合は 32.0%（481 名）であった。不正咬合を有する大学生では、正常咬合の者に比べて、ブラキシズムの自覚症状がある割合が有意に高かった（χ^2 検定、$P<0.01$）。ロジスティック回帰分析の結果では、男性において不正咬合の有無に関連する因子は、クレンチングの自覚（オッズ比 2.19、95%信頼区間 1.22-3.93、$P<0.01$）と低体重（BMI <18.5 kg/m^2）（オッズ比 1.89、95%信頼区間 1.31-2.71、$P<0.01$）であったが、女性ではそのような因子は認められなかった。不正咬合の種類別で分析した場合、男性では叢生とクレンチングの自覚および低体重との有意な関連があった（$P<0.01$）。

結論：男子大学生において、クレンチングの自覚および低体重は不正咬合（叢生）と関連があった。

・キーワード
ブラキシズム、不正咬合、若年者、横断研究
